# Urinary Measurement of Epigenetic DNA Modifications and 8-oxodG as Possible Noninvasive Markers of Colon Cancer Evolution

**DOI:** 10.3390/ijms232213826

**Published:** 2022-11-10

**Authors:** Aleksandra Skalska-Bugala, Agnieszka Siomek-Gorecka, Zbigniew Banaszkiewicz, Ryszard Olinski, Rafal Rozalski

**Affiliations:** 1 Department of Clinical Biochemistry, Faculty of Pharmacy, Collegium Medicum in Bydgoszcz, Nicolaus Copernicus University in Toruń, 85-092 Bydgoszcz, Poland; 2 Department of Surgery, Faculty of Medicine, Collegium Medicum in Bydgoszcz, Nicolaus Copernicus University in Toruń, 85-168 Bydgoszcz, Poland

**Keywords:** DNA demethylation, colon cancer, inflammatory bowel disease, adenoma, DNA epigenetic modification, 2D-UPLC-MS/MS, urine

## Abstract

The active DNA demethylation mechanism involves 5-methylcytosine (5-mCyt) enzymatic oxidation with the subsequent formation of 5-hydroxymethylcytosine (5-hmCyt), which can be further oxidized to 5-formylcytosine (5-fCyt) and 5-carboxylcytosine (5-caCyt). The products of active DNA demethylation are released into the bloodstream and eventually also appear in urine. We used online two-dimensional ultraperformance liquid chromatography with tandem mass spectrometry (2D-UPLC-MS/MS) to compare DNA methylation marks and 8-oxo-2′-deoxyguanosine (8-oxodG) in colorectal cancer and pre-cancerous condition in urine. The study included four groups of subjects: healthy controls, patients with inflammatory bowel disease (IBD), persons with adenomatous polyps (AD), and individuals with colorectal cancer (CRC). We have found that the level of 5-fCyt in urine was significantly lower for CRC and polyp groups than in the control group. The level of 5-hmCyt was significantly higher only in the CRC group compared to the control (2.3 vs. 2.1 nmol/mmol creatinine). Interestingly, we have found highly statistically significant correlation of 5-hydroxymethyluracil with 5-hydroxymethylcytosine, 5-(hydroxymethyl)-2′-deoxycytidine, 5-(hydroxymethyl)-2′-deoxyuridine, and 5-methyl-2′-deoxycytidine in the CRC patients’ group.

## 1. Introduction

Worldwide, colorectal cancer (CRC) is the third most common cancer in men and the second in women [1]. Clinical signs and symptoms vary depending on the location and the stage of the tumor and may not have symptoms in the early stages. CRC usually develops by neoplastic transformation of colon epithelial cells, giving rise to a benign polyp, which subsequently may progress to invasive carcinoma. More than 50% of CRC patients will develop metastasis, and approximately 25% of them present with metastasis at initial diagnosis [2]. Therefore, early-stage cancer detection is critical for reducing incidence and mortality.

Disruption of DNA epigenetic patterns can be an important factor of colon cancer early stage development. Environmental factors resulting in oxidative stress may be associated with epigenetic changes, and this may serve as an explanation of the observed differences in the incidence of colorectal cancer [3,4,5]. Cytosine methylation affects the cellular identity and organismal fate by gene repression [6]. The reverse of DNA methylation (demethylation) is equally important to activate previously silenced genes.

It is assumed that in this process, with the participation of ten-eleven translocation (TET) proteins, 5-methylcytosine (5-mCyt) is oxidized firstly to 5-hydroxymethylcytosine (5-hmCyt), and then to 5-formylcytosine (5-fCyt), which is probably converted to 5-carboxycytosine (5-caCyt) [7,8]. TET proteins together with cytosine deaminase can potentially lead to the synthesis of 5-hmUra, which is another compound with a potential role in the active demethylation of DNA [9,10,11].

In a recently published study we have found that the examined colonic pathologies have had their unique epigenetic marks, distinguishing them from each other, as well as from normal colonic tissue [12]. In this study we extend our work and compare DNA methylation marks in colorectal cancer and pre-cancerous condition in the urine of the different groups. This may lead to the identification of candidate epigenetic biomarkers for enhanced colorectal cancer risk assessment.

Up to now, the experiments have not focused on investigating the relationship between the effect of chronic inflammation on the generation of 5-hmCyt derivatives and their excretion with urine, even though inflammation-related metabolic pathways may influence the formation of 5-hmCyt and other compounds associated with the active demethylation pathway. In our study, we undertook the verification whether conditions predisposing to colorectal cancer, such as chronic inflammation as a direct consequence of inflammatory bowel disease (IBD), can shape DNA epigenetic modifications [13].

The outcome of IBD, which includes Crohn’s disease and ulcerative colitis, is a five-fold increased risk of colon cancer [14]. Chronic inflammation promotes carcinogenesis by inducing gene mutations, inhibiting apoptosis, and stimulating angiogenesis and cell proliferation. Inflammation also induces epigenetic alterations that are associated with cancer development [15]. Colorectal cancer is a major cause of death in both ulcerative colitis and colonic Crohn’s disease, accounting for 10 to 15% of all-cause mortality in inflammatory bowel disease. The risk of CRC increases with early age at IBD diagnosis, longer duration of symptoms and extent of the disease, with pancolitis having a more severe inflammation burden and risk of the dysplasia-carcinoma cascade [16]. Alterations in the normal DNA methylation may be involved in carcinogenesis, including tumor initiation, and some events are detectable before neoplastic transformation [17]. It was shown that epigenetic abnormalities can arise in the earliest steps of colorectal cancer development. Aberrant methylation patterns have been identified in preneoplastic lesions [18].

To the best of our knowledge no comprehensive study concerning measurement of urinary epigenetic DNA modifications have been reported up to date. Thus, using a recently developed, most reliable 2D-UPLC-MS/MS technique, we focus on the role of DNA epigenetic modifications in urine, in colon cancer development. Colonoscopy is still the gold standard for diagnostic tools for CRC detection. However, it is invasive and can be troublesome for the patient. Therefore, we would like to answer an important question as to whether the above-mentioned modifications might be noninvasive biomarkers of this process. Since colonic pathologies are associated with oxidative stress, aside from the epigenetic DNA modifications, we also analyzed the level of 8-oxo-2′-deoxy-7,8-dihydroguanosine (8-oxodG) as an established marker of this stress condition.

## 2. Results

**Figure 1:**
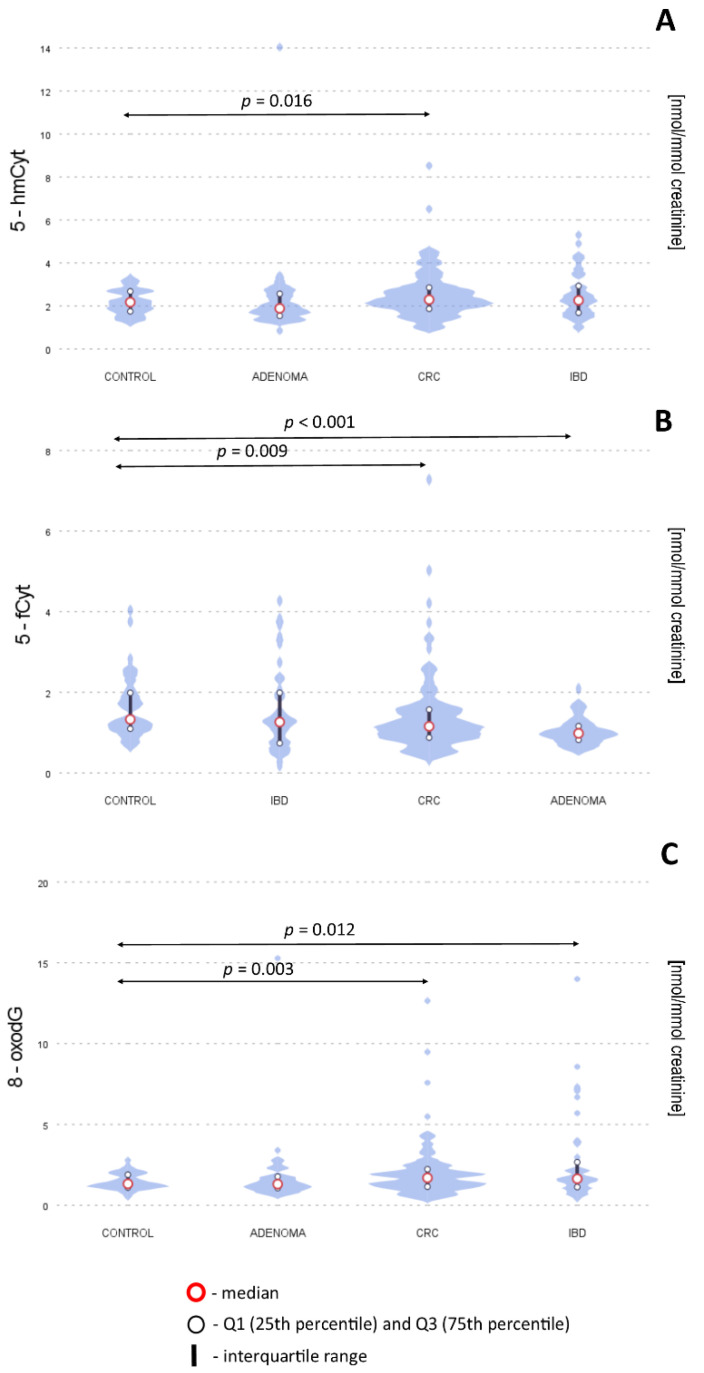
Levels of 5-hmCyt (**A**), 5-fCyt (**B**) and 8-oxodG (**C**) in urine from healthy controls, patients with adenomatous polyps, colorectal cancer (CRC) and inflammatory bowel disease (IDB). The results presented as medians and interquartile ranges. 5-hmCyt: 5-hydroxymethylcytosine; 5-fCyt: 5-formylcytosine; 8-oxodG: 8-oxo-2′-deoxyguanosine.

We have found that the level of 5-fCyt in urine was significantly lower for CRC and polyp groups than in the control group. The 8-oxodG excretion in the CRC and IBD subjects was significantly higher than in the control group (1.69 and 1.64 vs. 1.32 nmol/mmol creatinine). The level of 5-hmCyt was significantly higher only in the CRC group compared to the control group (Table 1 and Figure 1).

**Figure 2:**
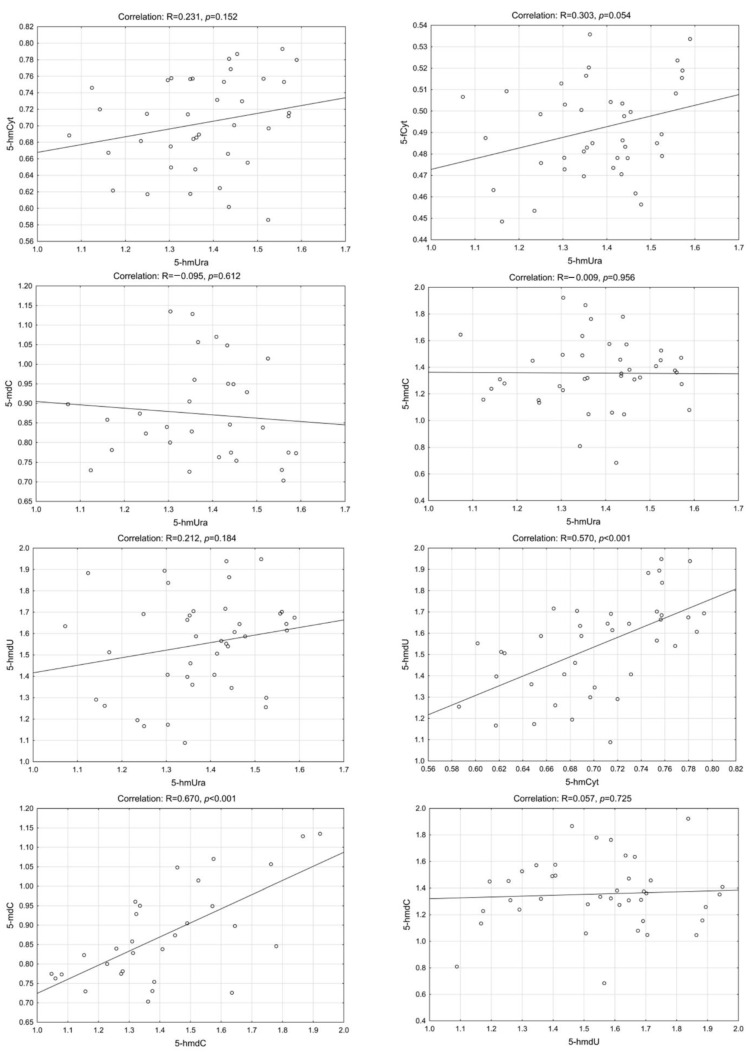
Selected correlations between the levels of DNA epigenetic modifications in urine—control group. 5-hmCyt: 5-hydroxymethylcytosine; 5-hmUra: 5-hydroxymethyluracil; 5-mdC: 5-methyl-2′-deoxycytidine; 5-hmdC: 5-(hydroxymethyl)-2′-deoxycytidine; 5-hmdU: 5-(hydroxymethyl)-2′-deoxyuridine; 5-fCyt: 5-formylcytosine.

**Figure 3:**
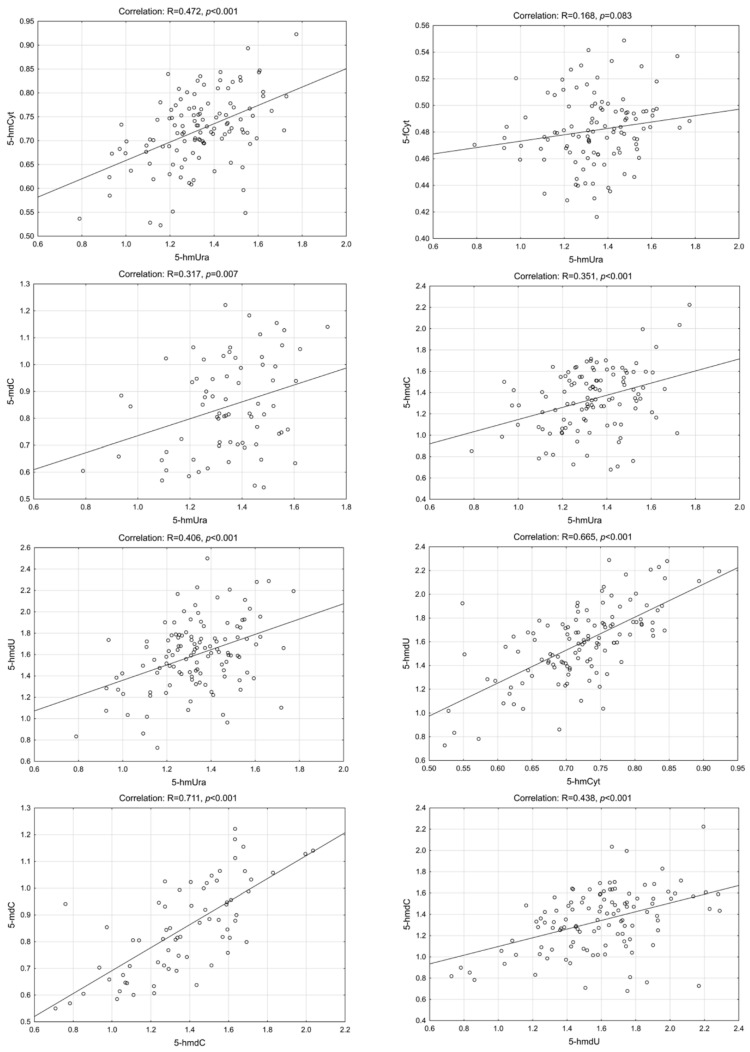
Selected correlations between the levels of DNA epigenetic modifications in urine—colorectal cancer group. 5-hmCyt: 5-hydroxymethylcytosine; 5-hmUra: 5-hydroxymethyluracil; 5-mdC: 5-methyl-2′-deoxycytidine; 5-hmdC: 5-(hydroxymethyl)-2′-deoxycytidine; 5-hmdU: 5-(hydroxymethyl)-2′-deoxyuridine; 5-fCyt: 5-formylcytosine.

**Figure 4:**
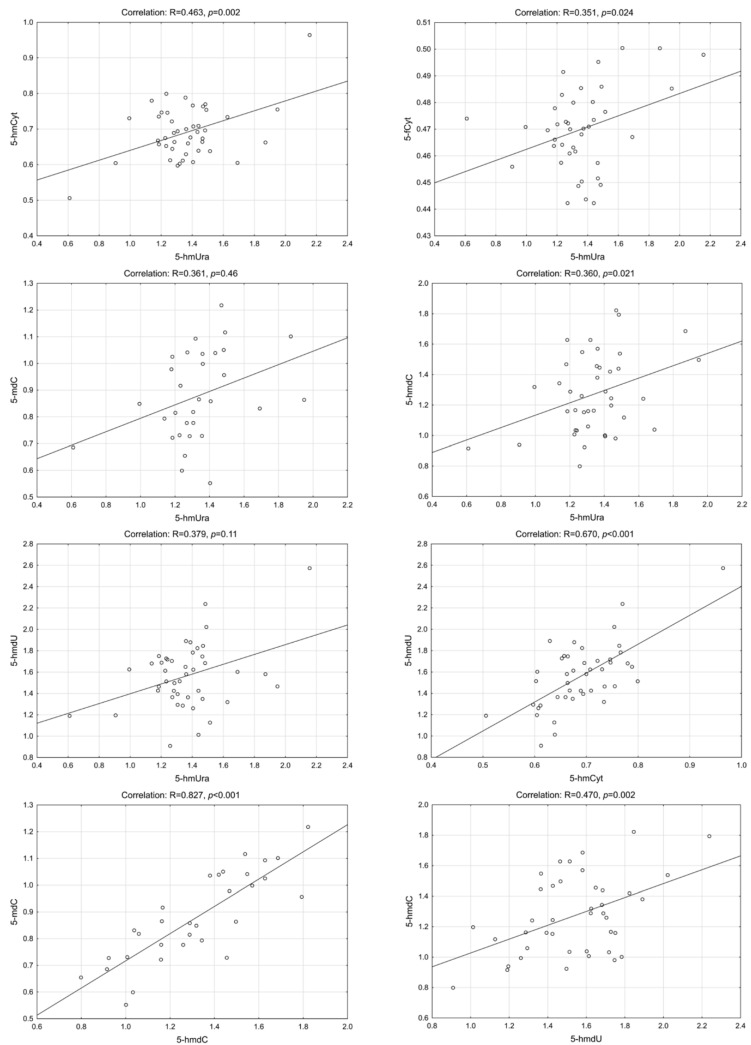
Selected correlations between the levels of DNA epigenetic modifications in urine—adenoma group. 5-hmCyt: 5-hydroxymethylcytosine; 5-hmUra: 5-hydroxymethyluracil; 5-mdC: 5-methyl-2′-deoxycytidine; 5-hmdC: 5-(hydroxymethyl)-2′-deoxycytidine; 5-hmdU: 5-(hydroxymethyl)-2′-deoxyuridine; 5-fCyt: 5-formylcytosine.

**Figure 5:**
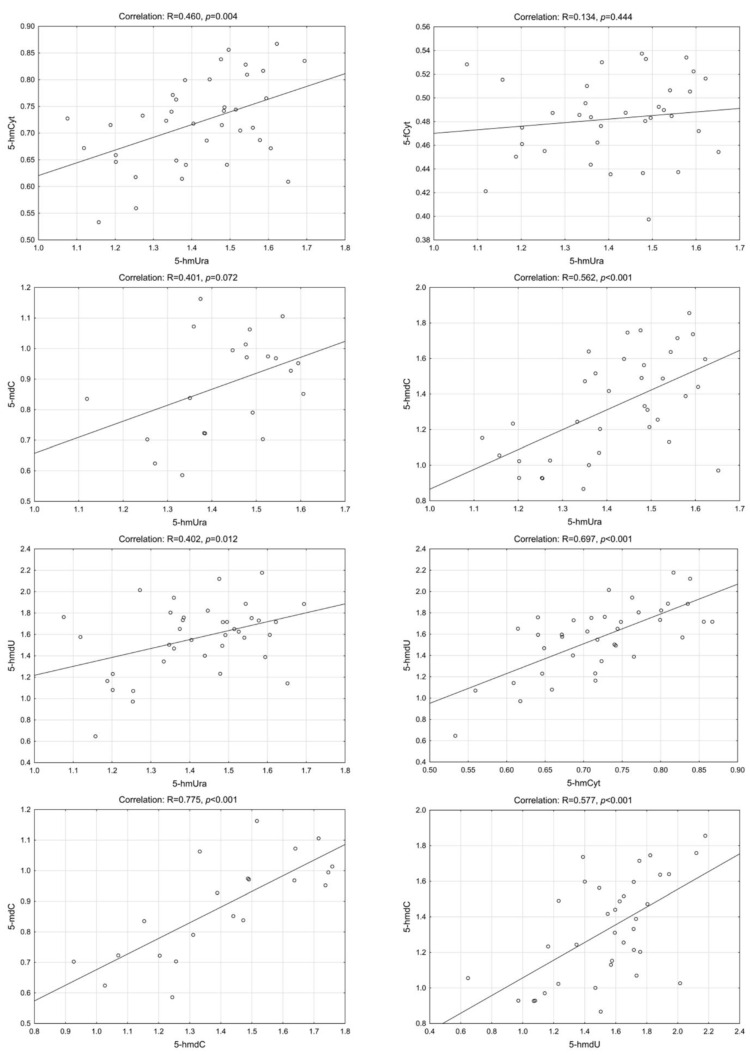
Selected correlations between the levels of DNA epigenetic modifications in urine—patients with inflammatory bowel disease (IDB). 5-hmCyt: 5-hydroxymethylcytosine; 5-hmUra: 5-hydroxymethyluracil; 5-mdC: 5-methyl-2′-deoxycytidine; 5-hmdC: 5-(hydroxymethyl)-2′-deoxycytidine; 5-hmdU: 5-(hydroxymethyl)-2′-deoxyuridine; 5-fCyt: 5-formylcytosine.

Interestingly, we have found a highly statistically significant correlation of 5-hmUra with 5-hmCyt, 5-hmdC, 5-mdC and 5-hmdU in the CRC patients group. Similar results were found in precancerous conditions (adenoma and IBD). The lowest correlation coefficients were observed for control/healthy subjects (Figure 2, Figure 3, Figure 4 and Figure 5—for more details see also Supplementary Materials: Tables S1–S5).

## 3. Discussion

The most plausible source of the epigenetic DNA modifications, as well as 8-oxodG, in urine, should be DNA repair. After the repair process is completed, modified bases and nucleosides are released into the bloodstream and, in the following step, are delivered into urine [19,20,21]. The whole-body epigenetic pattern can be evaluated, non-invasively, taking into account whole spectrum of urinary modifications, i.e., 5-hmCyt, 5-fCyt, 5-caCyt, 5-hmUra and their deoxynucleosides.

It is known that 5-fCyt and 5-caCyt may hinder DNA replication, which, in turn, may lead to genome instability and mutagenesis [22]. A specific set of enzymes, that effectively remove these modifications from DNA, was identified. Single-strand monofunctional uracil DNA glycosylase (SMUG1)) is the main enzyme involved in the removal of 5-hmUra from DNA and thymine DNA glycosylase (TDG) was demonstrated to exhibit a robust excision activity toward 5-fCyt or 5-caCyt in DNA [23,24,25]. Thus, the activity of the base excision repair pathways (BER) and processive demethylation may contribute to the presence of the modified bases/deoxynucleosides in urine.

Since only individual compounds were quantified in previous studies, we used 2D-UPLC-MS/MS to measure all the above-mentioned modifications in the same urine sample. Knowing that epigenetic changes may contribute significantly to carcinogenesis, we previously analyzed urinary levels of the modifications in two small groups: healthy controls and cancer patients. We found a significant difference in the urinary excretion of 5-hmdC between healthy subjects and CRC patients [19]. As mentioned above, 8-oxodG is the most widely recognized biomarker of oxidative stress and its urinary level is generally considered as marker of OGG1 (repair enzyme) activity [20]. Thus, the urinary excretion rate of this well-characterized moiety has been analyzed in our study as the reference point to levels of the epigenetic modifications.

Another efficient source of DNA epigenetic modifications in urine may be nucleotide salvage pathways. These pathways recycle deoxyribonucleosides that arise from the DNA breakdown or from cellular material ingested through the diet. Of note, rapidly proliferating cancer cells are dependent on an efficient supply of nucleotides, which is partially accomplished by salvage pathways that are upregulated in many cancers. However, the reincorporation of recycled modified nucleosides such as 5-hmdC may lead to deleterious effect for cells because they can alter gene expression. Therefore, they should be excluded from DNA incorporation by the selectivity of nucleotide kinases [26]. Fugger et al. described a metabolic pathway whereby cells eliminate 5-hmdCMP in a process requiring deamination of 5-hmCyt to 5-hmUra [22].

The above events should result in changes in the relationships between the levels of epigenetic DNA modifications and their derivatives in urine, therefore we decided to perform a correlation analysis. The key player in nucleotides salvage pathway as well as in processive demethylation pathway is 5-hmUra which originates from 5-hmCyt (see Section 1). As mentioned previously, upregulation of nucleotide salvage pathways enzymes was demonstrated in cancer cells [22,26]. It seemed to be reasonable that our observation might mirror gradual changes of the nucleotide salvage enzymes activity along the line IBD-adenoma-CRC. Recently, it was demonstrated that the presence of 5-hmUra in DNA can trigger the removal of adjacent molecules of 5-mCyt and 5-hmCyt by a mismatch repair system (MMR) and long-patch BER pathways (via processive demethylation) which, in turn, may explain the presence of 5-hmCyt and 5-mCyt bases/deoxynucleosides in urine and justify an excellent correlation between these modifications [27]. Moreover, the observed correlations between the modified bases/nucleosides could demonstrate their joint origin (via DNA repair systems mentioned above) and at the same time their participation in the same metabolic pathway (active DNA demethylation).

To conclude, our findings suggest that urinary levels of 5-fCyt and 8-oxodG may serve as noninvasive biomarkers of colorectal cancer development. Moreover, strong correlation of 5-hmUra with 5-hmCyt, 5-hmdC and 5-mdC supports recently published findings concerning 5-hmUra/5-hmdU as the key point in the nucleotide salvage pathway enzymes and, in turn, a determinant in cancer therapy [22]. Although the 2D-UPLC-MS/MS technique applied in our study is quite an expensive method and requires experienced staff, it is possible that semi-quantitative assays, based on commercially available antibodies, may be widely used in clinics for the detection of the aforementioned compounds.

## 4. Materials and Methods

The study included four groups of subjects: healthy controls (n = 45), patients with IBD (n = 42), persons with adenomatous polyps (n = 65), and individuals with colorectal cancer (n = 121). All of the study subjects were Caucasians. All participants of the study were recruited in a hospital setting (Collegium Medicum, Nicolaus Copernicus University, Bydgoszcz, Poland) and subjected to colonoscopy. The clinical details of the patients are presented in Table 2. Urine samples were collected before treatment began. The protocol of the study was approved by the Local Bioethics Committee, Collegium Medicum in Bydgoszcz, and Nicolaus Copernicus University in Torun (Poland). All the study participants provided written informed consent. Two-dimensional ultraperformance liquid chromatography with tandem mass spectrometry (2D-UPLC–MS/MS) was used for the epigenetic modification and 8-oxodG analysis of urine samples (with the exception of 5-hmUra).

### 4.1. The Determination of the Epigenetic Modifications and 8-oxodG Levels in Urine [28]

Two-dimensional ultra-performance liquid chromatography with tandem mass spectrometry (2D-UPLC–MS/MS) was used for the epigenetic modification analysis of the urine samples (with the exception of 5-hmUra). The urine samples were spiked with a mixture of internal standards at a 4:1 volumetric ratio. The 2D-UPLC−MS/MS system consists of a gradient pump and autosampler for one-dimensional chromatography, and a gradient pump and tandem quadrupole mass spectrometer with a UNISPRAY ion source was used for two-dimensional chromatography. Both systems were coupled with a column manager equipped with two programmable column heaters and two 2-position 6-port switching valves. The at-column dilution technique was used between the first and the second dimensions to improve the retention in the trap/transfer column. The sample molecules were then adsorbed to the packing material as very narrow bands that could be eluted with well-resolved, small-volume peaks. A diluting stream of water (0.5 mL/min) was pumped with a Waters 515 isocratic pump and mixed with the first-dimension column effluent using a UPLC low-dead-volume tee valve. The following columns were used: CORTECS UPLC T3 Column (1.6 µm, 3 mm × 150 mm) with a CORTECS T3 Van Guard precolumn (1.6 µm, 2.1 mm × 5 mm) for the first dimension, a Waters ACQUITY UPLC CSH C18 (1.7 µm, 2.1 mm × 100 mm) for the second dimension, and a Waters XSelect CSH C18 column (3.5 µm, 3 mm × 20 mm) as the trap/transfer column. The chromatographic system was operated in heart-cutting mode, which means that selected portions of effluent from the first dimension were loaded onto the trap/transfer column by 6-port valve switching, which served as an “injector” for the second dimension of the chromatography system. Mass spectrometric detection was conducted with a Waters Xevo TQ-S tandem quadrupole mass spectrometer equipped with a UniSpray ionization source. The following common detection parameters were used: source temperature, 150 °C; nitrogen desolvation gas flow, 1000 L/h; nitrogen cone gas flow, 150 L/h; desolvation temperature, 500 °C; and nebulizer gas pressure, 7 bar. Collision-induced dissociation was obtained with argon (6.0 at 3 × 10^−6^ bar pressure) as a collision gas. The instrument response to all compounds was optimized by the infusion of 10 µM genuine compounds dissolved in water (10 µL/min) in the mobile phase A stream via the mass spectrometer fluidics system operating in the “mixed” mode using the MassLynx 4.1 software IntelliStart feature. The quantitative and qualitative transition patterns and the specific settings of the detector are summarized in Table S6. The chromatographic system was operated with MassLynx 4.1 software from Waters. Quantitative analyses were performed using the TargetLynx application. All samples were analyzed with three to six technical replicates. Due to the low sensitivity of the method used, the level of 5-hmUra was determined by high-performance liquid chromatography for pre-purification followed by gas chromatography with isotope dilution mass spectrometric detection (LC/GC–MS), as previously described [29].

### 4.2. Statistical Analysis

The results are presented as median values, interquartile ranges and non-outlier ranges. Variables with a normal distribution were analyzed as “raw” data, whereas variables with non-normal distributions were subjected to Box–Cox transformation prior to statistical analyses based on parametric tests. The association between pairs of variables was analyzed based on Pearson correlation coefficients for raw or normalized data as applicable. All statistical transformations and analyses were carried out with Statistica 13.1 PL software [Dell Inc. (2016). Dell Statistica (data analysis software system), version 13. software.dell.com.]. The results were considered statistically significant at *p* values less than 0.05.

## Figures and Tables

**Table 1:** Urinary levels of DNA damage markers and active demethylation products of 5-methylcytosine.

[nmol/mmol Creatinine]	Control (N = 45)	CRC (N = 121)	AD (N = 65)	IBD (N = 42)	Control vs. CRC *p* Value	Control vs. AD *p* Value	Control vs. IBD *p* Value
**5-hmdU**	6.2 (4.6–7.4)	6.4 (4.5–8.3)	6.2 (4.4–7.7)	6.5 (4.6–8.3)	0.061	0.377	0.363
**5-mdC**	1.8 (1.2–3.2)	1.7 (0.82–4.0)	2.0 (1.0–5.1)	2.9 (1.0–4.0)	0.729	0.426	0.500
**5-hmCyt**	2.1 (1.7–2.6)	2.3 (1.8–2.8)	1.9 (1.5–2.4)	2.3 (1.7–3.0)	0.016 *	0.735	0.091
**5-fCyt**	1.3 (1.1–1.9)	1.1 (0.8–1.5)	1.0 (0.8–1.1)	1.3 (0.7–2.1)	0.009 *	<0.001 *	0.750
**5-hmdC**	2.1 (1.7–2.9)	2.3 (1.3–3.2)	1. 9 (1.1–2.8)	2.1 (1.2–3.4)	0.903	0.160	0.803
**8-oxodG**	1.3 (1.1–1.8)	1.7 (1.1–2.2)	1.3 (1.0–1.8)	1.6 (1.2–2.7)	0.003 *	0.368	0.012 *
**5-hmUra**	7.3 (5.8–8.2)	6.2 (5.0–8.2)	6.5 (6.1–8.3)	7.9 (6.3–9.9)	0.256	0.290	0.171

The results are presented as median values, and interquartile ranges (* *p* < 0.05). IBD—inflammatory bowel disease; AD –adenoma; CRC: colorectal cancer; 5-hmdU: 5-(hydroxymethyl)-2′-deoxyuridine; 5-mdC: 5-methyl-2′-deoxycytidine; 5-hmCyt: 5-hydroxymethylcytosine; 5-fCyt: 5-formylcytosine; 5-hmdC: 5-(hydroxymethyl)-2′-deoxycytidine; 8-oxodG: 8-oxo-7,8-dihydro-2′-deoxyguanosine; 5-hmUra: 5-hydroxymethyluracil. The urinary level of 5-carboxycytosine was under the limit of detection.

**Table 2:** Baseline characteristics of the study groups.

	Control (N = 45)	CRC (N = 121)	AD (N = 65)	IBD (N = 42)
Male (%)	44%	56%	59%	34%
Age (year)	56	65	64	34
Weight (kg)	77	75	80	61
Height (cm)	168	168	170	169
Body Mass Index	26.3	26.4	27.3	21.2
Histological Grade (%)		Stage: A—8% B—47% C—29% D—7% Hagitt scale I–IV—8%	AT—85% ATV—15%	

The results are presented as median values. AT—adenoma tubulare; ATV—adenoma tubulovillosum.

## Data Availability

The data that support the findings of this study are openly available inRepOD at https://doi.org/10.18150/PBI6JU.

## Supplementary Materials

The supporting information can be downloaded at: https://www.mdpi.com/article/10.3390/ijms232213826/s1.
