# Supplementary material for: Urinary Measurement of Epigenetic DNA Modifications and 8-oxodG as Possible Noninvasive Markers of Colon Cancer Evolution
Source: Int J Mol Sci. 2022 Nov 10;23(22):13826. doi: 10.3390/ijms232213826 (PMC9693012; doi:10.3390/ijms232213826)
Supplement: Supplementary file 1 [file ijms-23-13826-s001.zip › ijms-1954222-supplementary.pdf]

Urinary measurement of epigenetic DNA modifications and 8-oxodG as a possible noninvasive markers of colon cancer evolution.

Aleksandra Skalska-Bugala, Agnieszka Siomek-Gorecka, Zbigniew Banaszkiewicz, Ryszard Olinski and Rafal Rozalski

Supplementary Materials Page No.

Table S1. Correlation coefficients between the levels of DNA epigenetic modifications in urine - **control group**……………………………………………….….1

Table S2. Correlation coefficients between the levels of DNA epigenetic modifications in urine - **inflammatory bowel disease group** (IBD)……………..……2

Table S3. Correlation coefficients between the levels of DNA epigenetic modifications in urine - **colorectal cancer patients** …………………..…….…….…...3

Table S4. Correlation coefficients between the levels of DNA epigenetic modifications in urine - **precancerous patients (adenoma)**………..……………..…...4

Table S5. Correlation coefficients between the levels of DNA epigenetic modifications in urine for all analyzed cases ………………………….....……..…....5

Table S6. Transition patterns and specific detector settings for all compounds analyzed by 2D-UPLC–MS/MS in urine ……………………………….....…....6

Table S1. Correlation coefficients between the levels of DNA epigenetic modifications in urine - **control group**. Statistically significant correlations are indicated in red.

	5-hmdU	5-mdC	5-hmCyt	5-fCyt	5-hmdC	8-oxodG	5-hmUra
5-hmdU	R=1.0000<br>N=41<br>p= ---	R=-0.2364<br>N=31<br>p=0.200	R=0.5701<br>N=40<br>p<0.001	R=0.3642<br>N=41<br>p=0.019	R=0.0566<br>N=41<br>p=0.725	R=0.2600<br>N=41<br>p=0.101	R=0.2117<br>N=41<br>p=0.184
5-mdC	R=-0.2364<br>N=31<br>p=0.200	R=1.0000<br>N=31<br>p= ---	R=-0.2872<br>N=30<br>p=0.124	R=-0.2262<br>N=31<br>p=0.221	R=0.6698<br>N=31<br>p<0.001	R=0.1199<br>N=31<br>p=0.521	R=-0.0947<br>N=31<br>p=0.612
5-hmCyt	R=0.5701<br>N=40<br>p<0.001	R=-0.2872<br>N=30<br>p=0.124	R=1.0000<br>N=40<br>p= ---	R=0.3055<br>N=40<br>p=0.055	R=0.0326<br>N=40<br>p=0.842	R=0.2567<br>N=40<br>p=0.110	R=0.2309<br>N=40<br>p=0.152
5-fCyt	R=0.3642<br>N=41<br>p=0.019	R=-0.2262<br>N=31<br>p=0.221	R=0.3055<br>N=40<br>p=0.055	R=1.0000<br>N=41<br>p= ---	R=-0.0742<br>N=41<br>p=0.645	R=0.3079<br>N=41<br>p=0.050	R=0.3031<br>N=41<br>p=0.054
5-hmdC	R=0.0566<br>N=41<br>p=0.725	R=0.6698<br>N=31<br>p<0.001	R=0.0326<br>N=40<br>p=0.842	R=-0.0742<br>N=41<br>p=0.645	R=1.0000<br>N=41<br>p= ---	R=-0.0229<br>N=41<br>p=0.887	R=-0.0088<br>N=41<br>p=0.956
8-oxodG	R=0.2600<br>N=41<br>p=0.101	R=0.1199<br>N=31<br>p=0.521	R=0.2567<br>N=40<br>p=0.110	R=0.3079<br>N=41<br>p=0.050	R=-0.0229<br>N=41<br>p=0.887	R=1.0000<br>N=41<br>p= ---	R=0.3916<br>N=41<br>p=0.011
5-hmUra	R=0.2117<br>N=41<br>p=0.184	R=-0.0947<br>N=31<br>p=0.612	R=0.2309<br>N=40<br>p=0.152	R=0.3031<br>N=41<br>p=0.054	R=-0.0088<br>N=41<br>p=0.956	R=0.3916<br>N=41<br>p=0.011	R=1.0000<br>N=41<br>p= ---

Table S2.Correlation coefficients between the levels of DNA epigenetic modifications in urine - **inflammatory bowel disease group** (IBD). Statistically significant correlations are indicated in red.

	5-hmdU	5-mdC	5-hmCyt	5-fCyt	5-hmdC	8-oxodG	5-hmUra
5-hmdU	R=1.0000<br>N=38<br>p= ---	R=0.2532<br>N=21<br>p=0.268	R=0.6967<br>N=38<br>p<0.001	R=0.2508<br>N=35<br>p=0.146	R=0.5771<br>N=36<br>p<0.001	R=0.5006<br>N=38<br>p=0.001	R=0.4016<br>N=38<br>p=0.012
5-mdC	R=0.2532<br>N=21<br>p=0.268	R=1.0000<br>N=21<br>p= ---	R=0.1854<br>N=21<br>p=0.421	R=-0.0463<br>N=19<br>p=0.851	R=0.7745<br>N=21<br>p<0.001	R=0.1038<br>N=21<br>p=0.654	R=0.4011<br>N=21<br>p=0.072
5-hmCyt	R=0.6967<br>N=38<br>p<0.001	R=0.1854<br>N=21<br>p=0.421	R=1.0000<br>N=38<br>p= ---	R=0.3181<br>N=35<br>p=0.063	R=0.5124<br>N=36<br>p=0.001	R=0.4022<br>N=38<br>p=0.012	R=0.4596<br>N=38<br>p=0.004
5-fCyt	R=0.2508<br>N=35<br>p=0.146	R=-0.0463<br>N=19<br>p=0.851	R=0.3181<br>N=35<br>p=0.063	R=1.0000<br>N=35<br>p= ---	R=0.1442<br>N=34<br>p=0.416	R=0.0987<br>N=35<br>p=0.573	R=0.1337<br>N=35<br>p=0.444
5-hmdC	R=0.5771<br>N=36<br>p<0.001	R=0.7745<br>N=21<br>p<0.001	R=0.5124<br>N=36<br>p=0.001	R=0.1442<br>N=34<br>p=0.416	R=1.0000<br>N=36<br>p= ---	R=0.3146<br>N=36<br>p=0.062	R=0.5621<br>N=36<br>p<0.001
8-oxodG	R=0.5006<br>N=38<br>p=0.001	R=0.1038<br>N=21<br>p=0.654	R=0.4022<br>N=38<br>p=0.012	R=0.0987<br>N=35<br>p=0.573	R=0.3146<br>N=36<br>p=0.062	R=1.0000<br>N=38<br>p= ---	R=0.3713<br>N=38<br>p=0.022
5-hmUra	R=0.4016<br>N=38<br>p=0.012	R=0.4011<br>N=21<br>p=0.072	R=0.4596<br>N=38<br>p=0.004	R=0.1337<br>N=35<br>p=0.444	R=0.5621<br>N=36<br>p<0.001	R=0.3713<br>N=38<br>p=0.022	R=1.0000<br>N=38<br>p= ---

Table S3. Correlation coefficients between the levels of DNA epigenetic modifications in urine - **colorectal cancer patients**. Statistically significant correlations are indicated in red.

	5-hmdU	5-mdC	5-hmCyt	5-fCyt	5-hmdC	8-oxodG	5-hmUra
5-hmdU	R=1.0000<br>N=123<br>p= ---	R=0.3306<br>N=71<br>p=0.005	R=0.6651<br>N=119<br>p<0.001	R=0.1920<br>N=111<br>p=0.043	R=0.4375<br>N=114<br>p<0.001	R=0.1005<br>N=119<br>p=0.277	R=0.4055<br>N=118<br>p<0.001
5-mdC	R=0.3306<br>N=71<br>p=0.005	R=1.0000<br>N=72<br>p= ---	R=0.2763<br>N=68<br>p=0.023	R=0.1098<br>N=67<br>p=0.376	R=0.7105<br>N=68<br>p<0.001	R=-0.0005<br>N=68<br>p=0.996	R=0.3167<br>N=71<br>p=0.007
5-hmCyt	R=0.6651<br>N=119<br>p<0.001	R=0.2763<br>N=68<br>p=0.023	R=1.0000<br>N=119<br>p= ---	R=0.3846<br>N=107<br>p<0.001	R=0.3674<br>N=113<br>p<0.001	R=0.3657<br>N=118<br>p<0.001	R=0.4715<br>N=115<br>p<0.001
5-fCyt	R=0.1920<br>N=111<br>p=0.043	R=0.1098<br>N=67<br>p=0.376	R=0.3846<br>N=107<br>p=0.001	R=1.0000<br>N=112<br>p= ---	R=0.0195<br>N=102<br>p=0.845	R=0.2264<br>N=107<br>p=0.019	R=0.1676<br>N=108<br>p=0.083
5-hmdC	R=0.4375<br>N=114<br>p<0.001	R=0.7105<br>N=68<br>p<0.001	R=0.3674<br>N=113<br>p<0.001	R=0.0195<br>N=102<br>p=0.845	R=1.0000<br>N=114<br>p= ---	R=0.1042<br>N=113<br>p=0.272	R=0.3511<br>N=110<br>p<0.001
8-oxodG	R=0.1005<br>N=119<br>p=0.277	R=-0.0005<br>N=68<br>p=0.996	R=0.3657<br>N=118<br>p<0.001	R=0.2264<br>N=107<br>p=0.019	R=0.1042<br>N=113<br>p=0.272	R=1.0000<br>N=119<br>p= ---	R=0.2026<br>N=115<br>p=0.030
5-hmUra	R=0.4055<br>N=118<br>p<0.001	R=0.3167<br>N=71<br>p=0.007	R=0.4715<br>N=115<br>p<0.001	R=0.1676<br>N=108<br>p=0.083	R=0.3511<br>N=110<br>p<0.001	R=0.2026<br>N=115<br>p=0.030	R=1.0000<br>N=122<br>p= ---

Table S4.Correlation coefficients between the levels of DNA epigenetic modifications in urine - **precancerous patients (adenoma)**. Statistically significant correlations are indicated in red.

	5-hmdU	5-mdC	5-hmCyt	5-fCyt	5-hmdC	8-oxodG	5-hmUra
5-hmdU	R=1.0000<br>N=44<br>p= ---	R=0.3000<br>N=31<br>p=0.101	R=0.6702<br>N=44<br>p<0.001	R=0.2160<br>N=41<br>p=0.175	R=0.4703<br>N=41<br>p=0.002	R=0.2699<br>N=44<br>p=0.076	R=0.3790<br>N=44<br>p=0.011
5-mdC	R=0.3000<br>N=31<br>p=0.101	R=1.0000<br>N=31<br>p= ---	R=0.0368<br>N=31<br>p=0.844	R=0.1185<br>N=29<br>p=0.540	R=0.8273<br>N=31<br>p<0.001	R=0.1121<br>N=31<br>p=0.548	R=0.3614<br>N=31<br>p=0.046
5-hmCyt	R=0.6702<br>N=44<br>p<0.001	R=0.0368<br>N=31<br>p=0.844	R=1.0000<br>N=44<br>p= ---	R=0.3514<br>N=41<br>p=0.024	R=0.4026<br>N=41<br>p=0.009	R=0.4082<br>N=44<br>p=0.006	R=0.4632<br>N=44<br>p=0.002
5-fCyt	R=0.2160<br>N=41<br>p=0.175	R=0.1185<br>N=29<br>p=0.540	R=0.3514<br>N=41<br>p=0.024	R=1.0000<br>N=41<br>p= ---	R=0.1747<br>N=38<br>p=0.294	R=0.2329<br>N=41<br>p=0.143	R=0.3513<br>N=41<br>p=0.024
5-hmdC	R=0.4703<br>N=41<br>p=0.002	R=0.8273<br>N=31<br>p<0.001	R=0.4026<br>N=41<br>p=0.009	R=0.1747<br>N=38<br>p=0.294	R=1.0000<br>N=41<br>p= ---	R=0.2986<br>N=41<br>p=0.058	R=0.3595<br>N=41<br>p=0.021
8-oxodG	R=0.2699<br>N=44<br>p=0.076	R=0.1121<br>N=31<br>p=0.548	R=0.4082<br>N=44<br>p=0.006	R=0.2329<br>N=41<br>p=0.143	R=0.2986<br>N=41<br>p=0.058	R=1.0000<br>N=44<br>p= ---	R=0.3835<br>N=44<br>p=0.010
5-hmUra	R=0.3790<br>N=44<br>p=0.011	R=0.3614<br>N=31<br>p=0.046	R=0.4632<br>N=44<br>p=0.002	R=0.3513<br>N=41<br>p=0.024	R=0.3595<br>N=41<br>p=0.021	R=0.3835<br>N=44<br>p=0.010	R=1.0000<br>N=44<br>p= ---

Table S5.Correlation coefficients between the levels of DNA epigenetic modifications in urine for all analyzed cases. Statistically significant correlations are indicated in red.

	5-hmdU	5-mdC	5-hmCyt	5-fCyt	5-hmdC	8-oxodG	5-hmUra
5-hmdU	R=1.0000<br>N=248<br>p= ---	R =0.2568<br>N=156<br>p=0.001	R=0.6616<br>N=243<br>p<0.001	R=0.2044<br>N=230<br>p=0.002	R=0.4254<br>N=234<br>p<0.001	R=0.2232<br>N=244<br>p<0.001	R=0.3612<br>N=243<br>p<0.001
5-mdC	R=0.2568<br>N=156<br>p=0.001	R =1.0000<br>N=157<br>p= ---	R=0.1502<br>N=152<br>p=0.065	R=0.0191<br>N=148<br>p=0.818	R=0.7331<br>N=153<br>p<0.001	R=0.0596<br>N=153<br>p=0.464	R=0.2881<br>N=156<br>p<0.001
5-hmCyt	R=0.6616<br>N=243<br>p<0.001	R=0.1502<br>N=152<br>p=0.065	R=1.0000<br>N=243<br>p= ---	R=0.3299<br>N=225<br>p<0.001	R=0.3713<br>N=232<br>p<0.001	R=0.3834<br>N=242<br>p<0.001	R=0.4180<br>N=239<br>p<0.001
5-fCyt	R=0.2044<br>N=230<br>p=0.002	R=0.0191<br>N=148<br>p=0.818	R=0.3299<br>N=225<br>P<0.001	R=1.0000<br>N=231<br>p= ---	R=0.0517<br>N=217<br>p=0.449	R=0.1871<br>N=226<br>p=0.005	R=0.1925<br>N=227<br>p=0.004
5-hmdC	R=0.4254<br>N=234<br>p<0.001	R=0.7331<br>N=153<br>p<0.001	R=0.3713<br>N=232<br>p<0.001	R=0.0517<br>N=217<br>p=0.449	R=1.0000<br>N=234<br>p= ---	R=0.1605<br>N=233<br>p=0.014	R=0.3280<br>N=230<br>p<0.001
8-oxodG	R=0.2232<br>N=244<br>p<0.001	R=0.0596<br>N=153<br>p=0.464	R=0.3834<br>N=242<br>p<0.001	R=0.1871<br>N=226<br>p=0.005	R=0.1605<br>N=233<br>p=0.014	R=1.0000<br>N=244<br>p= ---	R=0.2748<br>N=240<br>p<0.001
5-hmUra	R=0.3612<br>N=243<br>p<0.001	R=0.2881<br>N=156<br>P<0.001	R=0.4180<br>N=239<br>p<0.001	R=0.1925<br>N=227<br>p=0.004	R=0.3280<br>N=230<br>p<0.001	R=0.2748<br>N=240<br>p<0.001	R=1.0000<br>N=247<br>p= ---

Table S6. Transition patterns and specific detector settings for all compounds analyzed by 2D UPLC–MS/MS in urine.

compound name		ionization mode	nominal molecular mass (Da)	pseudo-molecular ion formulation	nominal parent ion (Da)	nominal daughter ion	capillary (kV)	cone (V)	collision (eV)
**5-hydroxymethylcytosine**	quantifier	UniSpray+	141	[M+H]$^+$	142	81	1.3	40	18
	qualifier	UniSpray+	141	[M+H]$^+$	142	124	1.3	40	12
**[D_3]-5-hydroxymethylcytosine**	quantifier	UniSpray+	144	[(M+3)+H]$^+$	145	84	1.3	40	18
	qualifier	UniSpray+	144	[(M+3)+H]$^+$	145	127	1.3	40	12
**5-hydroxymethyl-2'-deoxycytidine**	quantifier	UniSpray+	257	[M+H]$^+$	258	124	1.3	35	22
	qualifier	UniSpray+	257	[M+H]$^+$	258	142	1.3	35	10
**[D_3]-5-hydroxymethyl-2'-deoxycytidine**	quantifier	UniSpray+	260	[(M+3)+H]$^+$	261	127	1.3	35	22
	qualifier	UniSpray+	260	[(M+3)+H]$^+$	261	145	1.3	35	10
**5-carboxylcytosine**	quantifier	UniSpray-	155	[M-H]$^-$	154	67	1.3	60	12
	qualifier	UniSpray-	155	[M-H]$^-$	154	110	1.3	60	12
**[$^{13}C_{10}$, $^{15}N_2$]-5-carboxylcytosine**	quantifier	UniSpray-	162	[(M+7)-H]$^-$	161	71	1.3	60	12
	qualifier	UniSpray-	162	[(M+7)-H]$^-$	161	116	1.3	60	12
**8-oxo-2'-deoxyguanosine**	quantifier	UniSpray+	283	[M+H]$^+$	284	168	1.3	30	18
	qualifier	UniSpray+	283	[M+H]$^+$	284	140	1.3	30	18
**[$^{15}N_5$]-8-oxo-2'-deoxyguanosine**	quantifier	UniSpray+	288	(M+5)+H+	289	173	1.3	20	15
	qualifier	UniSpray+	288	(M+5)+H+	289	145	1.3	20	15
**5-formylcytosine**	quantifier	UniSpray+	139	[M-H]$^-$	140	97	1.3	40	15
	qualifier	UniSpray+	139	[M-H]$^-$	140	70	1.3	40	15
**[$^{13}C_{10}$, $^{15}N_2$]-5-formylcytosine**	quantifier	UniSpray+	146	[(M+7)-H]$^-$	147	102	1.3	40	15
	qualifier	UniSpray+	146	[(M+7)-H]$^-$	147	73	1.3	40	15
**5-methyl-2'-deoxycytidine**	quantifier	UniSpray+	241	[M-H]$^-$	242	126	1.3	30	12
	qualifier	UniSpray+	241	[M-H]$^-$	242	224	1.3	30	12
**[$^{13}C_{10}$, $^{15}N_2$]-5-methyl2'-deoxycytidine**	quantifier	UniSpray+	253	[(M+12)-H]$^-$	254	133	1.3	30	12
	qualifier	UniSpray+	241	[(M+12)-H]$^-$	254	236	1.3	30	12
